# Engineering bacteria to control electron transport altering the synthesis of non-native polymer†

**DOI:** 10.1039/d1ra06403g

**Published:** 2021-12-21

**Authors:** Mechelle R. Bennett, Akhil Jain, Katalin Kovacs, Phil J. Hill, Cameron Alexander, Frankie J. Rawson

**Affiliations:** Division of Regenerative Medicine and Cellular Therapies, Biodiscovery Institute, School of Pharmacy, University of Nottingham University Park Nottingham NG7 2RD UK Frankie.Rawson@nottingham.ac.uk; Synthetic Biology Research Centre, School of Life Sciences, University of Nottingham University Park, Nottingham NG7 2RD UK; Division of Microbiology, Brewing and Biotechnology, School of Bioscience, University of Nottingham Sutton Bonington Campus Nottingham LE15 5RD UK; Division of Molecular Therapeutics and Formulation, Boots Science Building, School of Pharmacy, University of Nottingham University Park Nottingham NG7 2RD UK

## Abstract

The use of bacteria as catalysts for radical polymerisations of synthetic monomers has recently been established. However, the role of trans Plasma Membrane Electron Transport (tPMET) in modulating these processes is not well understood. We sort to study this by genetic engineering a part of the tPMET system NapC in *E. coli*. We show that this engineering altered the rate of extracellular electron transfer coincided with an effect on cell-mediated polymerisation using a model monomer. A plasmid with arabinose inducible PBAD promoters were shown to upregulate NapC protein upon induction at total arabinose concentrations of 0.0018% and 0.18%. These clones (*E. coli*_(IP_0.0018%)_ and *E. coli*_(IP_0.18%)_, respectively) were used in iron-mediated atom transfer radical polymerisation (Fe ATRP), affecting the nature of the polymerisation, than cultures containing suppressed or empty plasmids (*E. coli*_(IP_S)_ and *E. coli*_(E)_, respectively). These results lead to the hypothesis that EET (Extracellular Electron Transfer) in part modulates cell instructed polymerisations.

## Abstract 2

The use of bacteria as catalysts for radical polymerisations of synthetic monomers has recently been established.

## Graphical abstract

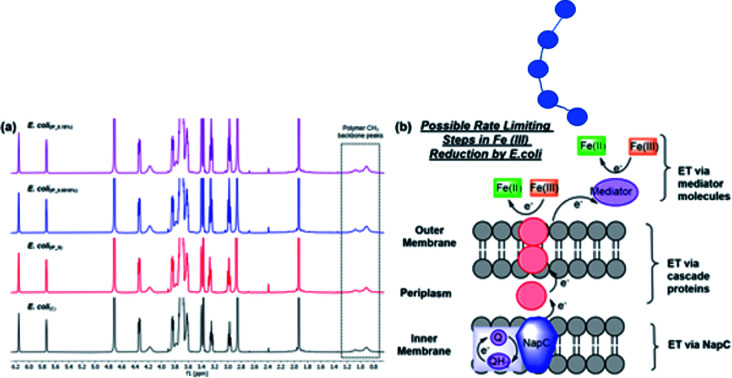

## 1. Introduction

Electron transfer plays a fundamental role in modulating biology, enabling almost all cellular functions.^1^ Transplasma membrane electron transport systems [tPMETs] are essential from a biological energetic perspective and for mediation of cell redox and signaling.^2,3^ It has recently been shown that electron transfer *via* tPMETs can be used to synthesise biopolymers (defined as polymers synthesised by cells) and may have a role in radical polymerisations of synthetic monomers.^4–8^ This was achieved by tPMET modulating the redox state of metal ions facilitating atom transfer radical polymerisation or redox-mediated oxidative polymerisation events. Importantly, the fabrication of *in situ* generated biopolymers *via* microbes, opens new strategies for interfacing cells with new materials and may find applications controlling cell behaviour and connecting cellular bioelectrical relays and improving application in microbial fuel cells.^8–12^ To date however, the underlying cellular redox systems that modulate polymerisation have not been studied in depth. One area that is expected to modulate the synthesis of polymers is bacterial electron transfer, however, there is a lack of understanding of how biologically derived electron transfer affects polymer synthesis.

One approach to controlling bacterial electron transfer is to adopt synthetic biology protocols to tune bacterial behaviour.^13^ Consequently, we were inspired to explore a synthetic biology approach to tune electron transfer from bacteria for Fe ATRP under aqueous conditions. It has been previously shown that electrogenic *Cupriavidus metallidurans* (*C. met*)^14–17^ and *Escherichia coli* (*E. coli*) are able to start Fe ATRP reactions.^4^ Metal ion reduction takes place *via* microbial external electron transfer (EET) involving membrane bound C type cytochrome (C-Cyt) proteins, possibly in combination with synergistic Fe homeostasis efflux pumps.^9^ To further understand the role of microbial EET in Fe catalysis and resulting polymerisation, C-Cyt (NapC of *E. coli*) was our engineering target. C-Cyt was chosen as a target to mediate cell instructed ATRP as cytochromes are known to play a substantial role in bacterial EET that is required for energy production, particularly involving the reduction of metal ions.^18–21^ The electron transport chain of *S. oneidensis MR1* is well researched and the corresponding C-Cyts can contribute to metal ion reduction,^22,23^ with protein CymA integral to the reduction of iron.^24^ NapC is also a member of the NapC/NirT family of C-Cyt, existing in the periplasmic membrane of *E. coli*.^25^ NapC showed ferric reductase activity like its homologue, CymA, with the ability to substitute for CymA when inserted into *Shewanella* strains.^26^ As *E. coli* K12 has been previously shown to initiate ATRP with Cu-,^7^ and Fe-catalysts,^4^ it was sought as a vehicle to investigate the effect of EET on polymerisation by altering NapC levels. To study the resulting change in EET we used an electrochemical method that we have recently reported.^2,27^ Herein we find that overexpression of the NapC protein in *E. coli* alters EET and this changes the kinetics of the reported cell instructed bio-mediated polymerisations.

## 2. Methods

### 2.1 Materials

All chemicals were purchased from the supplier and used without further purification unless stated. Iron(ii) chloride hydrate FeCl_2_·4H_2_O and l-arabinose were purchased from Sigma Aldrich. Iron(iii) chloride hexahydrate (FeCl_3_·6H_2_O) ≥98% was purchased from Scientific Laboratory supplies. Ascorbic acid (AscA) >99% was purchased from Alfa Aesar. For bacteria growth lysogeny broth (LB) was used. GenElute™ bacterial genomic DNA kit was purchased from Sigma-Aldrich. Monarch® plasmid miniprep kit, Monarch® DNA gel extraction kit and Monarch® PCR & DNA cleanup kit was purchased from New England Biolabs (NEB). Pierce™ BCA protein assay kit was purchased from Thermo Fisher Scientific. Polyethylene glycol methacrylate (PEGMA) and 2-hydroxyethyl 2-bromoisobutyrate (ATRP inhibitor; HEBIB) were purchased from Sigma, UK.

*napC* gene sequence

ATGGGAAATTCTGACCGTAAGCCTGGTCTGATTAAGCGCCTGTGGAAATGGTGGCGTACCCCCAGCCGTCTGGCGCTGGGGACGCTGCTGTTGATCGGTTTTGTTGGCGGCATCGTCTTCTGGGGTGGCTTTAACACCGGGATGGAAAAAGCCAATACCGAAGAGTTCTGCATTAGCTGCCACGAAATGCGCAACACGGTGTATCAGGAATACATGGATTCCGTGCACTACAACAACCGTAGCGGCGTCCGTGCGACCTGTCCGGATTGTCACGTTCCGCACGAGTTTGTGCCGAAGATGATACGCAAGCTCAAAGCAAGTAAAGAGCTGTATGGTAAAATTTTTGGCGTTATTGACACGCCGCAGAAATTTGAAGCTCATCGTCTGACGATGGCACAGAATGAGTGGCGGCGCATGAAGGACAATAACTCGCAGGAGTGCCGTAACTGTCACAACTTCGAGTATATGGATACAACCGCC CAGAAATCGGTTGCCGCGAAGATGCATGACCAGGCGGTGAAAGATGGGCAAAC CTGTATTGATTGCCATAAAGGGATAGCGCACAAGCTGCCCGATATGCGTGAAGTCGAGCCAGGTTTTTAA (sourced using Kegg genome database for *Escherichia coli* K-12 MG1655: b2202, https://www.genome.jp/dbget-bin/www_bget?eco:b2202).

### 2.2 Bacteria, plasmid and primers

Bacterial strains, plasmids and primers used in this work are listed in Tables S1 and S2,† respectively.

### 2.3 Storage, growth conditions and transformation

Bacterial cultures were stored at – 80 °C on beads from Microbank™ long term bacterial storage system (Prolabs Diagnostics). For recovery of cultures, *E. coli* top 10 wild type were grown from beads at 37 °C overnight (at a fixed 18 hours for all cultures) in Lysogeny Broth (LB; 5 mL) with agitation. *E. coli* harbouring pMTL8000 series plasmids with Cm resistance gene (*catP*) were grown from beads at 30 °C overnight (18 hours) in LB (5 mL) and 2.5 μL Cm stock solution with agitation. The chemical transformation of complete plasmids into competent cells was carried out according to the NEB chemical transformation protocol. Chemically competent cells were thawed for 10 minutes on ice and then 15 μL of plasmid DNA was added. The mixture was incubated on ice for 30 minutes before being heat shocked at 42 °C for exactly 30 seconds. Afterwards, they were placed on ice for another 5 minutes and later 500 μL SOC (Super Optimal broth with Catabolite repression) media (NEB) was added before incubating at 37 °C for 60 minutes. After this, the cells were spread on agar plates (with chloramphenicol, Cm) and left to grow at 24 °C for 4 days.

### 2.4 Construction of inducible promoter vector containing for NapC overexpression

For overexpression of NapC, pMTL83153_P_BAD__NapC vector containing an inducible promoter P_BAD_ upstream of *napC* was generated. Firstly, *napC* region was isolated from *E. coli* gDNA and amplified through PCR. During the next step, an arabinose inducible promoter (P_BAD__*araC*) region was obtained through PCR amplification from the plasmid pMTL71101_P_BAD__araC using P_BAD__araC_fwd and P_BAD__araC_rev primers. Next, the plasmid pMTL83153 was digested with restriction enzymes Not1 and Sal1 to remove the P_fdx_ constitutive promoter. Finally, Hifi ligation assembly was carried out to insert the P_BAD__araC and napC region into the pMTL83153_(-P_fdx_) vector to obtain pMTL83153_P_BAD__NapC inducible promoter vector.

### 2.5 Colony PCR and sequencing

Colony PCR (primers: ColE1+tra_F2 and pCB102_R1) was conducted to determine napC insertion into pMTL83153_P_BAD__NapC inducible promoter vector. Colony PCR amplified DNA regions were sequenced using sanger sequencing (Source Bioscience service) and results analysed using Benchling sequence alignment tool.

### 2.6 NapC expression and quantification

Clones containing the *P*_*BAD*_ promoter were induced using l-arabinose according to manufacturer protocol in manual ‘pBAD/His A, B, and C pBAD/Myc-His A, B, and C’ (Invitrogen™). Briefly, a culture of the clone was grown in 5 mL LB with 2.5 μL Cm stock at 30 °C overnight. A total of 3 tubes containing 10 mL LB were labelled and 5 μL culture was added to achieve OD_600 nm_ 0.05. These were grown to OD_600 nm_ 0.5 and arabinose (0.1 mL, Table S3†) was added to each. After 8 hours (or stationary phase achieved), 2 mL of each culture was harvested by centrifugation (8000 rpm, 10 minutes, 4 °C) and stored at −20 °C overnight. The total protein content was evaluated using Pierce™ BCA protein assay kit (Thermo Scientific™) according to the manufacturer's instructions. A BSA standard sample was analysed for protein content using the BCA assay at different concentrations to create a standard curve (Fig. S2†). A standard curve was used to compare the absorbance of protein from cell lysates to calculate total protein concentration in each sample. SDS-PAGE was performed to confirm the expression of NapC.

### 2.7 Arabinose toxicity

Cultures were grown overnight in 5 mL of LB with 2.5 μL Cm. These were adjusted to 0.05 OD in 2 mL LB (+Cm) and grown to OD_600_ 0.4. Finally, the cultures were induced with arabinose at a predetermined concentration as shown in Table S3.†

### 2.8 Fe atom transfer radical polymerisation (ATRP)

*E. coli*_(IP)_ cultures were grown and induced according to protocol mentioned in earlier section (NapC expression and quantification). The induced clones were grown to OD_600_ ∼1.1 (20 mL LB + 10 μL Cm), centrifuged (6000 rpm, 20 min), washed using PBS and then resuspended in degassed PBS (1.5 mL, approximately 1.35 × 10^10^ CFU mL^−1^ in final reaction volume (4 mL)) before using in the Fe ATRP reactions. For each reaction an appropriate volume of FeCl_3_·6H_2_O and Me_6_TREN (Table S5†) were added to a 5 mL Eppendorf in PBS under stirring. Alongside in separate tubes, polyethylene glycol methacrylate (PEGMA) and 2-hydroxyethyl 2-bromoisobutyrate (ATRP inhibitor; HEBIB) were dissolved in PBS. The above mixture was placed in the anaerobic cabinet for >1 hour to allow degassing. After degassing, bacteria were added to FeCl_3_/Me_6_TREN mix and pre-mixed for 15 minutes before adding a monomer/initiator mixture. The reaction was left overnight and terminated by exposure to air. The kinetics of each reaction were monitored by ^1^H NMR and the resulting polymer were analysed with Size Exclusion Chromatography (SEC).

### 2.9 Electrochemical studies

Linear Sweep Voltammetry (LSV) analyses were carried out using a 3-electrode system: carbon fibre micro-disk working electrode (33 μm), Ag/AgCl reference electrode and platinum wire counter electrode from ALS Co. Ltd, Japan. Experiments were conducted using potentiostat ‘Autolab PGStat302A’ with low current module (EDC). Measurements and analysis were carried out using NOVA 2.1 software. All electrochemical experiments were carried out in 1× phosphate buffer saline solution (PBS) supporting electrolyte, at current range 100 pA, scan rate 100 mV s^−1^ from 1.25 V to −0.25 V at room temperature.

### 2.10 Calibration curve for Fe reduction

For the calibration curve, LSV of potassium ferricyanide and ferrocyanide (1 mM) mixed in different ratios (10 : 0, 8 : 2, 5 : 5, 2 : 8, 0 : 10) was performed in PBS (10 mM). A sample of PBS was applied to all measurements as a control baseline subtraction. A PK-3 electrode polishing kit (ALS Co. Ltd) was used to polish the working electrode between measurements. The first derivative functions of the calibration voltammograms were taken to create a cross-referencing tool to determine steady state reduction (*I*_ss,red_), steady state oxidation (*I*_ss,ox_) peaks and extract the rate of change (d(*I*_ss_)/d*t*). The concentration of the redox analyte was calculated using Randles–Sevcik equation. The steady state current (*I*_ss_) is measured:*I*_ss_ = 4*nFDCr*where, *n* = number of electrons, *F* = Faradays constant (6485.3 C mol^−1^), *D* = diffusion coefficient (cm^2^ s^−1^), *C* = analyte concentration (mol cm^−3^) and *r* = radius of microelectrode (cm).

Finally, the calibration graph was created by plotting the d(*I*_ss_)/d*t* peak values against ferricyanide or ferrocyanide concentrations. The lines of best fit could then be compared to first derivative values of LSV graphs for samples reduced by bacterial.

### 2.11 ^1^H NMR

^1^H NMR spectra were recorded at room temperature on a 400 MHz (Bruker DPX400 Ultrashield) using deuterated solvents (D_2_O). NMR spectra were analysed using MestReNova 11.0.0-17609 2016 Mestrelab Research S.L.

### 2.12 Size exclusion chromatography (SEC)

THF SEC was performed Shimadzu prominence LC-20AD system equipped with 2× Agilent PLgel Mixed-D columns heated to 40 °C. Samples were eluted with THF + 2% TEA + 0.01% BHT (flow rate 1 mL min^−1^) over 30 min. Injection vol. was set to 50 μL. *M*_n_ and *Đ* were calculated using PMMA standards (InfinityLab EasiVial) with MWs ranging from 600–1.5 million g per mol.

### 2.13 Electrochemical determination of Fe^3+^

Bacteria were induced/grown overnight (10 mL, LB + Cm) and pellets were resuspended in potassium ferricyanide (5 mL, 1 mM) for 1 hour at 37 °C. Bacteria were removed by centrifugation (6000 rpm, 10 minutes) and the supernatant was analysed by LSV. Prior to incubation with ferricyanide clones were either (i) suppressed by addition of glucose *E. coli*_(IP_S)_, (ii) activated by 0.0018% total arabinose concentration *E. coli*_(IP_0.0018%)_ or (ii) activated by 0.18% total arabinose concentration *E. coli*_(IP_0.18%)_. Three aliquots of each supernatant sample were scanned (*n* = 3) using LSV. The whole experiment was repeated twice with new biological samples. The first derivative of the voltammograms was taken and Fe concentrations were calculated using the calibration graph.

## 3. Results and discussion

**Fig. 1:**
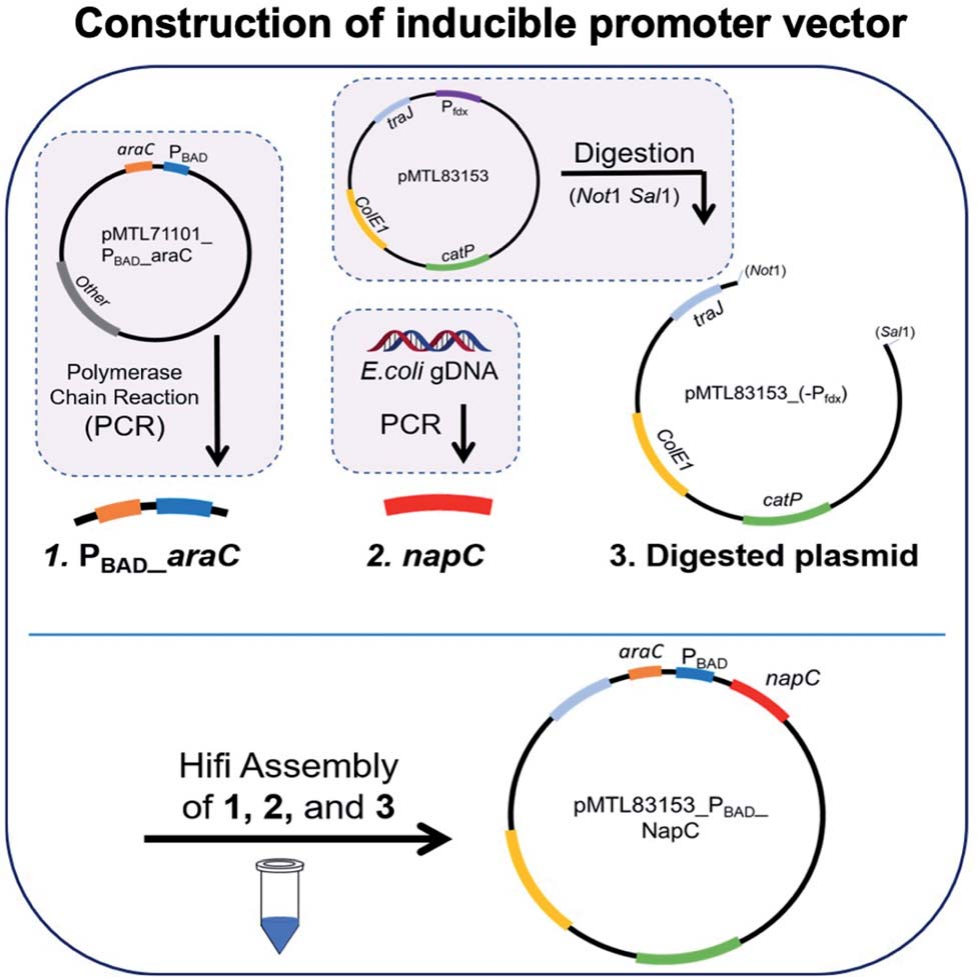
Assembly of inducible promoter vector containing promoter P_BAD_ for NapC overexpression and control. PCR was carried out with specific primers to extract and amplify regions (1) P_BAD__araC and (2) napC. Region 1 was obtained from the plasmid pMTL71101_P_BAD__araC and region 2 was obtained from *E. coli* gDNA. The plasmid pMTL83153 was digested with REs *Not*I and *Sal*I to remove the Pfdx promoter, resulting in region 3, digested plasmid. The three regions were ligated together with Hifi assembly to create the completed vector. Gel electrophoresis of colony PCR products for DNA regions making up the inducible promoter vector, against 1 KB Plus DNA ladder P_BAD__araC DNA region (1278 bps) and napC DNA region (633 bps) are as expected. Sanger sequencing diagrams for inducible promoter vector showing matching DNA regions of sequencing with forward and reverse primers compared to a model sequence.

We initially took a synthetic biology approach to rewire tPMET behaviour. We began by cloning *napC* gene isolated from *E. coli* into the plasmid pMTL83153 (details are given in Table S1†), immediately downstream from the non-constitutive inducible promoter (P_BAD_). We chose P_BAD_ because high quantities of transcribed protein (NapC) were desired to facilitate investigations into its effect on iron reduction which is indicative of EET. P_BAD_ would also allow us to control any potential toxicity and NapC expression by optimising the concentration of inducer (arabinose). To achieve this, a pMTL8000 modular plasmid collection was designed for ease of component selection during cloning. This can also be useful for tuning *via* plasmid replicons (controls replication efficiency), markers (antibiotic resistance selection), promoters (drives the transcription of the target gene), and multiple cloning sites (MCSs) (containing restriction sites for restriction enzyme cloning).^28^ Although these were created to aid cloning in *Clostridium* cultures, they are hosted in *E. coli* and so were convenient in the cloning of *napC* into *E. coli*. The plasmid constructed, based on pMTL83153 (Fig. 1a), confers resistance to chloramphenicol^29^ and *napC* under the control of PBAD.

Plasmid pMTL83153 was assembled using the *napC* gene and P_BAD_ promoter region, amplified from *E. coli* and plasmid pMTL71101_P_BAD__araC, respectively, amplified and purified. These *napC* and P_BAD_ regions were inserted in *Not* and *Sal*I digested pMTL83153. The transformation of the cloned vectors led to the growth of colonies that were selected on Cm plates and screened by colony PCR (primers: ColE1+tra_F2 and pCB102_R1; Table S2†). Gel electrophoresis was used to analyse the resultant amplicons and confirm the successful insertion of P_BAD__araC DNA region (1278 bps) and *napC* DNA region (633 bps) (Fig. 1b). Finally, the authenticity of the cloned pMTL83153_P_BAD__NapC inducible promoter vector colonies (*E. coli*_(IP)_) was confirmed using Sanger sequencing of the amplified DNA fragments (Fig. 1c) and designated pBADNAP.

After the successful cloning of *napC*, we examined the resulting NapC protein expression by *E. coli*. alongside clones harbouring a control plasmid, pMTL83151, referred to as the ‘empty plasmid’ (*E. coli*_(E)_). NapC expression in bacteria containing pBADNAP (*E. coli*_(BADNAP)_) was induced by the addition of arabinose, which binds to AraC, activating the P_BAD_ promoter and initiates transcription of the protein. This expression was tuned by optimising the arabinose concentration (Table S3†) and its effect on NapC content was examined by performing SDS-PAGE (Fig. S1†). *E. coli*_(BADNAP)_ were exposed to a final arabinose concentration of 0% (band I_0_), 0.000018% (band I_1_), 0.0018% (band I_2_), and 0.18% (band I_3_). As a control measure, arabinose was also added to *E. coli*_(E)_ (band E), permitting a consistent comparison between protein expression levels. The total protein quantification was analysed using bicinchoninic acid (BCA) protein assay (Fig. S2 and Table S4†). A certain band of interest was identified in the resulting protein gel (Fig. S1 and S3,† circled green area).^25^ From the BCA assay the maximum expression was found when at 0.00018% inducer was used.

**Fig. 2:**
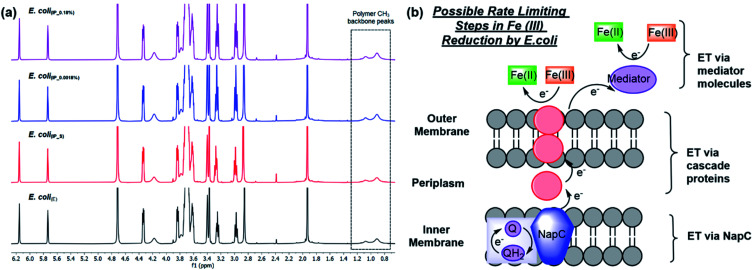
Fe ATRP polymerisations activated by *E. coli* cultures harbouring different plasmids to compare the effects of NapC protein upregulation. (a) ^1^H NMR spectra of Fe ATRP activated by *E. coli*_(E)_ (black), or inducible promoter plasmids, *E. coli*_(IP)_ either (i) suppressed by addition of glucose *E. coli*_(IP_S)_ (red), (ii) activated by 0.0018% total arabinose concentration *E. coli*_(IP_0.0018%)_ (blue) or (ii) activated by 0.18% total arabinose concentration *E. coli*_(IP_0.18%)_ (purple). (b) Possible rate limiting steps in Fe(iii) reduction including electron transfer (ET) *via* NapC, ET *via* cascade proteins and ET *via* mediator molecules.

As shown previously, Fe ATRP can be activated by bacteria including *E. coli*.^4^ To investigate the effects of changing NapC expression on Fe reduction rates, *E. coli*_(BADNAP)_ were used in Fe ATRP reactions (Table S5†) alongside *E. coli*_(E)_. An iron catalysed ATRP of the water-soluble monomer poly(ethylene glycol)methyl ether methacrylate (PEGMA, *M*_n_ = 300 gmol @ 1) was carried out at 37 °C with tris(2-dimethylaminoethyl)amine (Me_6_TREN) for Fe^3+^ reduction. The kinetics of each reaction was monitored by ^1^H NMR (Fig. 2a) and the resulting polymers were analysed by SEC (Fig. S4† and Table 1). A larger polymer yield for *E. coli*_(BADNAP_0.0018%)_ and *E. coli*_(BADNAP_0.18%)_ activated reactions was observed, compared to those activated by *E. coli*_(E)_ or *E. coli*_(BADNAP_S)_ cultures. Importantly, all cell polymerisation studies were performed when cells had reached an OD of 500 and therefore observed effects were not due to differing growth rates. This suggested that the upregulation of the NapC protein had some effect on the rates of Fe^3+^ reduction to Fe^2+^. As this difference is small, it suggests that there could be other factors contributing to EET from the bacteria to the Fe catalyst. These might include electron transfer (ET) across the periplasm *via* other cascade proteins in the electron transport chain, or EET between shuttle molecules and the Fe catalyst (Fig. 2b).

**Table 1:** Fe ATRP polymerisations activated by *E. coli* cultures harbouring different plasmids to compare the effects of NapC protein upregulation

Culture	Conversion	*M*_n_^th^ (kDa)	*M*_n_^SEC^ (kDa)	*Đ*[*c*]
*E. coli*_(E)_	38.4%	23.3	251.9	3
*E. coli*_(IP_S)_	38.8%	23.5	237.4	2.9
*E. coli*_(IP_0.0018%)_	46.5%	28.1	228	2.7
*E. coli*_(IP_0.18%)_	45.6%	27.6	230	3

To note the *Đ*[*c*] values suggest the polymerisation is not very well controlled. However, we do show bioelectrical activity is one factor affecting the mechanism of polymerisation and will be discussed in more detail in the proceeding experiments. However, more research is required to deconstruct the other biological controlling factors.

**Fig. 3:**
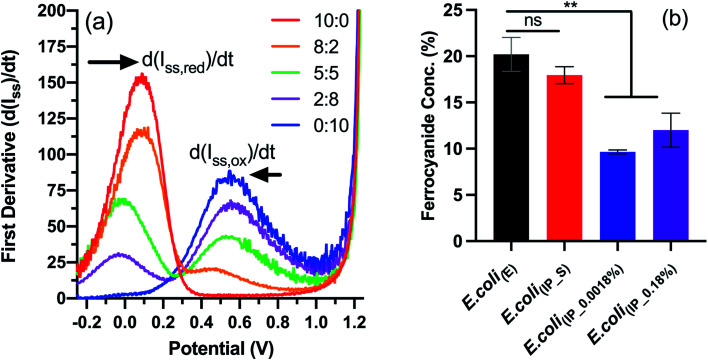
Electrochemical detection of ferrocyanide using linear sweep voltammetry. (a) First derivative function applied to linear sweep voltammogram of ferricyanide/ferrocyanide redox couple (Fig. S6†) to determine d(*I*_ss_)/d*t* values. (b) Concentrations (%) of ferrocyanide detected in the supernatant of samples incubated for 1 hour with *E. coli*_(IP)_ or *E. coli*_(E)_. Results are expressed as mean ± S.D. ** *P* < 0.005 *vs. E. coli*_(E)_, obtained using 1-way ANOVA with a Dunnett's post-test. LSV was used to analyse the supernatant of incubated samples (*N* = 2, *n* = 6) and the first derivative function was applied to resulting voltammograms. The concentrations were determined using the calibration graph showed in Fig. S7.†

We next wanted to study the effect on electron transfer when altering the NapC expression levels of the tPMET. This was achieved using linear sweep voltammetry (LSV), an electrochemical technique that reports on the concentration of ferricyanide and ferrocyanide (Fe reduction), which is indicative of the electron transfer rate when measured over time. We have previously reported this LSV based method to detect concentration changes corresponding to EET in cells.^27,30,31^ In brief, the first derivative function of the LS voltammograms (Fig. S5†) obtained from known concentrations of ferricyanide/ferrocyanide redox couple was used to calculate the rate of change in steady-state current (d(*I*_ss_)/d*t*) (Fig. 3a). A calibration curve of concentration *vs.* (d(*I*_ss_)/d*t*) (Fig. S6†) was generated to determine whether *E. coli*_(IP)_ clones with upregulated NapC protein could reduce more Fe^3+^ in the form of ferricyanide. Next, the samples of *E. coli*; *E. coli*_(E)_, *E. coli*_(IP_S)_ (suppressed by addition of glucose to inhibit P_BAD_ activation of NapC expression),^32^*E. coli*_(IP_0.0018%)_, and *E. coli*_(IP_0.18%)_ were incubated with ferricyanide for 1 hour at 37 °C, after which the bacteria were removed by centrifugation and the supernatant was taken and LSV was performed on the supernatant. The average current was taken for each sample and the first derivative graph was plotted to determine d(*I*_ss_)/d*t* values. Finally, the d(*I*_ss_)/d*t* values of each sample were compared to the calibration graph and the concentration of ferrocyanide (Fe^2+^) in solution was plotted as a percentage of total Fe concentration.

The data indicate that *E. coli*_(E)_ and *E. coli*_(IP_S)_ cultures reduced more ferricyanide to ferrocyanide than the *E. coli*_(IP)_ clones (Fig. 3b). This is of note because higher ferricyanide reduction was expected from *E. coli* clones that were induced with arabinose as indicated by the western blot which indicated increased NapC expression when compared to the cells modified with the control plasmid (Fig. S3†). We attribute this to a shift in the bioenergetics of the bacteria, that could have been triggered in *E. coli*_(IP_0.0018%)_ and *E. coli*_(IP_0.18%)_ cultures, whereby EET reduction pathways were stunted due to the use of excess energy required for the over-production of the NapC protein. Interestingly, there was a slight increase in the ferricyanide reduction with *E. coli*_(IP_0.18%)_ cultures compared to *E. coli*_(IP_0.0018%)_ cultures. We show arabinose induction as 0.18% slowed the growth of *E. coli*_(IP)_ (Fig. S7†). This increased stress in *E. coli*_(IP_0.18%)_ cultures could have triggered EET systems (such as NapC) increasing ferricyanide reduction. Stress induced tPMET upregulation pathways were also observed in analogous experiments with other cell types,^2^ suggesting that cells utilise EET systems to balance bioenergetic requirements. Although the bioenergetics of Fe metabolism in living organisms remains challenging to study,^33^ further studies are underway to gain more insights through monitoring Fe reducing behaviour in stress-induced environments, such as temperature, pH and chemical treatments.

## 4. Conclusions

In summary, the cloning of the *napC* gene into *E. coli* was achieved and controlled with the P_BAD_ promoter. We established that there were differences in electron transfer rates dependent on the arabinose concentration. The nature of polymerisation for ATRP catalysed by *E. coli*_(IP_0.0018%)_ and *E. coli*_(IP_0.0018%)_ were different, suggesting that NapC regulation has some effect on the Fe^3+^ reduction system. This subsequently affects the nature of the polymerisation. On the modification of NapC levels, there appear to be alterations in extracellular electron transfer which coincides with variations in differences in polymerisation kinetics.

## Author contributions

Conceptualization: M. R. B., F. J. R., C. A. P. J. H., data curation: M. R. B., F. J. R., C. A. and K. K. Formal analysis: M. R. B., F. J. R., and K. K. Funding acquisition: F. J. R. Investigation: M. R. B. Methodology: M. R. B., F. J. R. and C. A. Project administration: F. J. R. Resources: F. J. R., C. A., P. J. H., K. K. Supervision: C. A., P. J. H., F. J. R. Validation: M. R. B., A. J., C. A., F. J. R. Visualization: F. J. R., M. R. B. and C. A. Writing: A. J., M. R. B. and F. J. R. Writing – review & editing: A. J., M. R. B., F. J. R., C. A., P. J. H., K. K.

## Conflicts of interest

There are no conflicts to declare.

## Supplementary Material

RA-012-D1RA06403G-s001
