# Supplementary material for: Engineering bacteria to control electron transport altering the synthesis of non-native polymer
Source: RSC Adv. 2021 Dec 21;12(1):451–7. doi: 10.1039/d1ra06403g (PMC8978702; doi:10.1039/d1ra06403g)
Supplement: RA-012-D1RA06403G-s001 [file RA-012-D1RA06403G-s001.pdf]

Electronic Supplementary Material (ESI) for RSC Advances.
This journal is © The Royal Society of Chemistry 2021

Supporting Information

Engineering Bacteria to Control Electron Transport Altering the Synthesis of Biopolymer

Mechelle R. Bennett, [a] *Akhil Jain,* [a, b] *Katalin Kovacs,* [b] *Phil J. Hill,* [c] *Cameron Alexander,* [d] *Frankie J. Rawson** [a]

[a] Division of Regenerative Medicine and Cellular Therapies, Biodiscovery Institute, School of Pharmacy, University of Nottingham, University Park, Nottingham, NG7 2RD

*E-mail: Frankie.Rawson@nottingham.ac.uk

[b] Synthetic Biology Research Centre, School of Life Sciences, University of Nottingham, University Park, Nottingham, NG7 2RD

[c] Division of Microbiology, Brewing and Biotechnology. School of Bioscience, University of Nottingham, Sutton Bonington Campus, Nottingham LE15 5RD

[d] Division of Molecular Therapeutics and Formulation, Boots Science Building, School of Pharmacy, University of Nottingham, University Park, Nottingham, NG7 2RD

Table S1. Bacteria and Plasmid Strains.

Strain/Plasmid	Description	Source/Reference
Escherichia coli wild type	Plasmid Storage Strain (K12 Top 10)	Invitrogen
pMTL_83153	Modular plasmid containing pCB102, catP, ColE1 + tra, P_{fdx}+ MCS	http://www.clostron.com/pMTL80000.php

Table S2. Oligonucleotide Primers used for PCR of DNA regions. All Primers used for PCR with Q5 ® Polymerase. *Exceptions used for Colony PCR with Green DreamTaq.

Primers	Sequence (5'-3')	Tm (°C)	Function
NapC_fwd_hifi	GAGCGAAATCATGGGAA ATTCTGACCGTAAG	61.8	To amplify *napC* in cloning with **P_{BAD} promoter**
NapC_rev_hifi	ATCTCCATGGACGCGTG ACGTTAAAAACCTGGCTC GAC	59.3	To amplify *napC* in cloning with **P_{BAD} promoter**
P_{BAD}_araC-fwd	CAGGAAACAGCTATGAC CGCTTATGACAACTTGAC GGC	59.3	Amplify **P_{BAD} promoter**
P_{BAD}_araC_rev	AATTTCCCATTTTCTCCT CTTTAATCTAGAGAATTC	58.9	Amplify **P_{BAD} promoter**
ColE + *tra*_F2*	CCATCAAGAAGA GCGAC	56.7	**Colony PCR**
pCB102_R1*	GATAGTCAAAAGGCATAA CAG	55.4	**Colony PCR**

Table S3. Arabinose induction concentrations for *E. coli* containing Inducible Promoter Vector.

Sample	Stock arabinose (w/v)	Final arabinose Concentration

E. coli$_{(IP\_0\%)}$	0	0
E. coli$_{(IP\_0.000018\%)}$	0.0018%	0.000018%
E. coli$_{(IP\_0.0018\%)}$	0.18%	0.0018%
E. coli$_{(IP\_0.18\%)}$	18%	0.18%

Table S4. Protein standardisation calculations using BCA assay. *Calculated using BSA equation (y=0.0016x). **Note samples were diluted by 2; therefore, the concentrations were doubled to obtain original solution concentrations.

**Vector**	**Abs$_{562nm}$ (av)**	**Sample conc (µg/mL)***	**Original conc (µg/mL)****	**mg/ml**
Empty Plasmid	1.57	982.7	1965	1.97
E.coli$_{(IP\_0\%)}$	1.63	1019	2038	2.04
E.coli$_{(IP\_0.000018\%)}$	1.69	1053	2106	2.11
E.coli$_{(IP\_0.0018\%)}$	1.65	1029	2058	2.06
E.coli$_{(IP\_0.18\%)}$	1.40	873	1746	1.75

Table S5. Reagent ratios for *E.coli*$_{IP}$ initiated Fe ATRP.

**Reagent**	**Ratio**	**Mmol**	**Mass (mg)**	**Vol (uL)**
PEGMA	100	0.052	17	16.2
$FeCl_3.6H_20$	4.65	0.0024	0.65	-
Me_6TREN	13.95	0.0072	1.7	1.9
HEBIB	2	0.0010	0.22	0.15

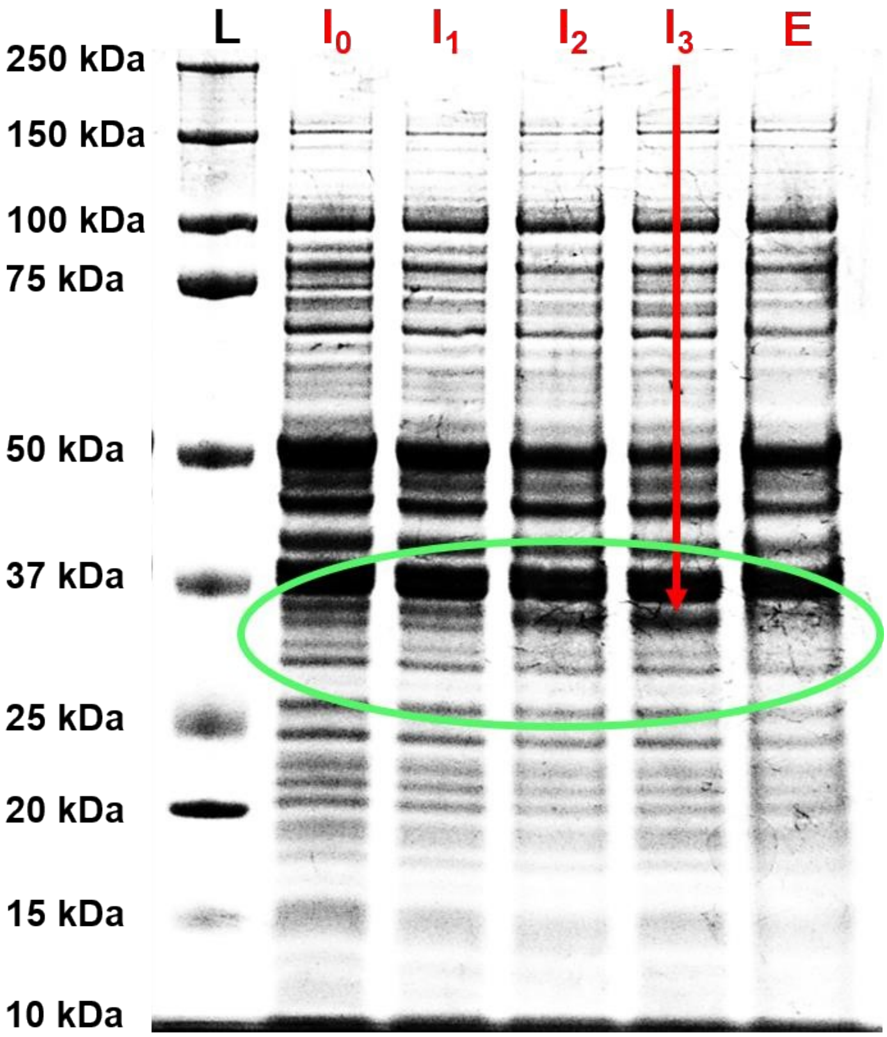

Figure S1. Protein expression analysis. SDS PAGE Gel for lysates of bacteria containing empty plasmid (E), Inducible promoter vector with 0% (I_0), 0.000018% (I_1), 0.0018% (I_2) and 0.18% (I_3) total arabinose concentration induction. Protein Gel against Precision Plus Protein™ Kaleidoscope ladder (L).

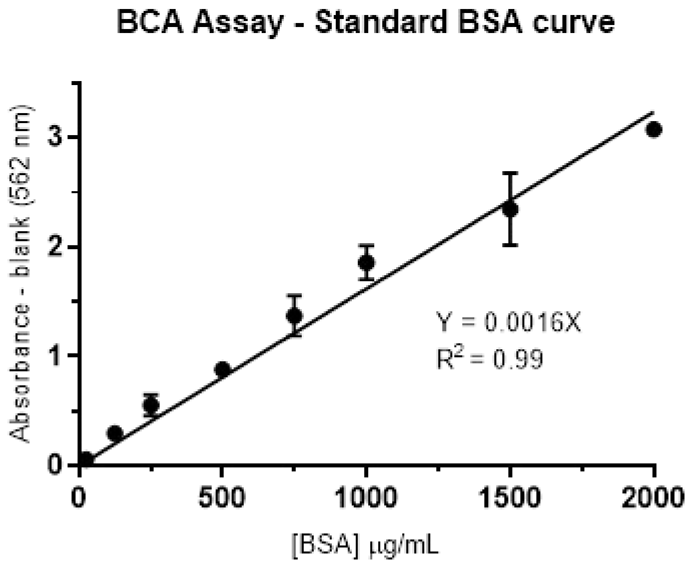

Figure S2. BCA Standard curve for BSA (Bovine Serum albumin).

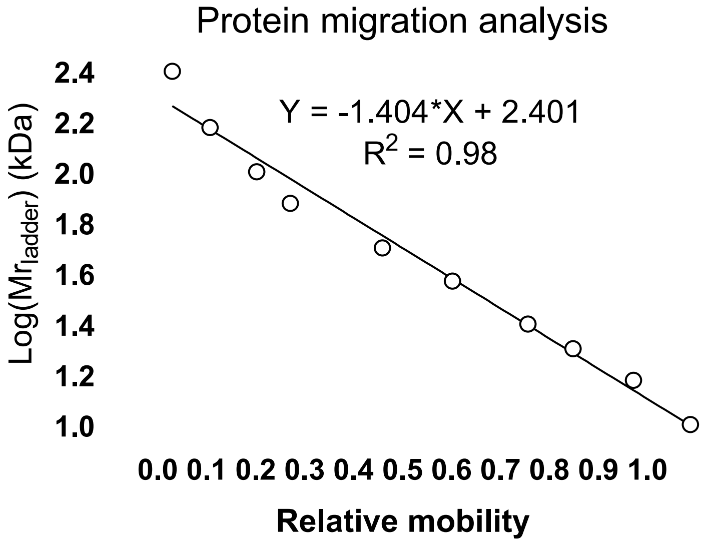

Figure S3. Protein migration analysis of protein ladder Log(Mr) against relative mobility on SDS PAGE Gel.

Calculation:

Migration (NapC) = 10.7 cm Dye

front = 17.2 cm

$$\text{Relative mobility (X)} = \frac{\text{migration}}{\text{dye front}} = \frac{10.7}{17.2} = 0.622$$

$$Log(Mr_{NapC}) = [-(1.404 * X) + 2.401]$$

$$Log(Mr_{NapC}) = (-1.404 * 0.622) + 2.401$$

$$Mr_{NapC} = 10^{((-1.404*0.622)+2.401)} = 33.7\ kDa$$

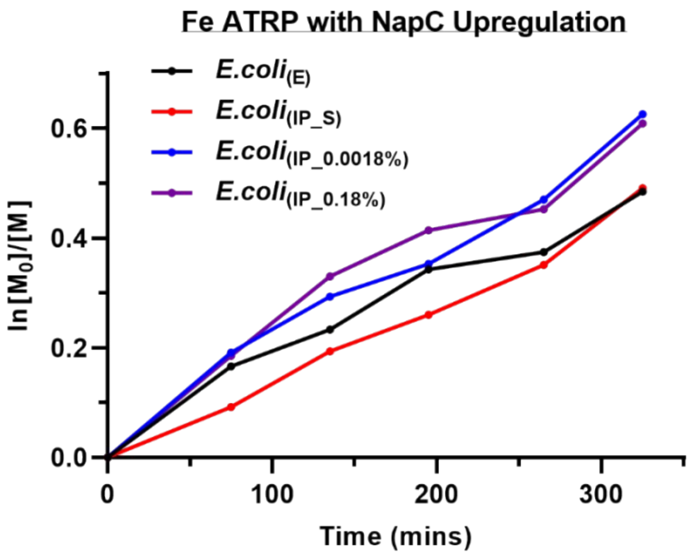

Figure S4. ^{1}H NMR kinetics of Fe ATRP activated by *E. coli* harbouring empty plasmids, *E. coli*$_{(E)}$, or inducible promoter plasmids, *E. coli*$_{(IP)}$ either i) suppressed by addition of glucose *E. coli*$_{(IP\_S)}$, ii) activated by 0.0018% total arabinose concentration *E. coli*$_{(IP\_0.0018\%)}$ or ii) activated by 0.18% total arabinose concentration *E. coli*$_{(IP\_0.18\%)}$.

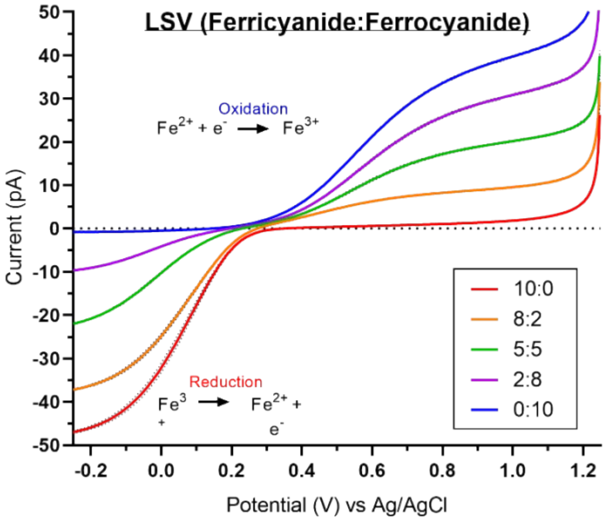

Figure S5. Linear sweep voltammogram of Current Vs Potential carried out using 3 electrode system with carbon fibre micro-disk electrode (33 μm), Ag/AgCl reference electrode and Pt counter electrode in 1X PBS electrolyte. Scans were carried out at 100 mV/s from 1.25 V to -0.25 V. 1 mM potassium ferricyanide and ferrocyanide were made in PBS (1X) and mixed in ratios (10:0, 8:2, 5:5, 2:8, 0:10) and voltammograms observed (n=3, error = SD) for each sample, where electrode was polished between each scan.

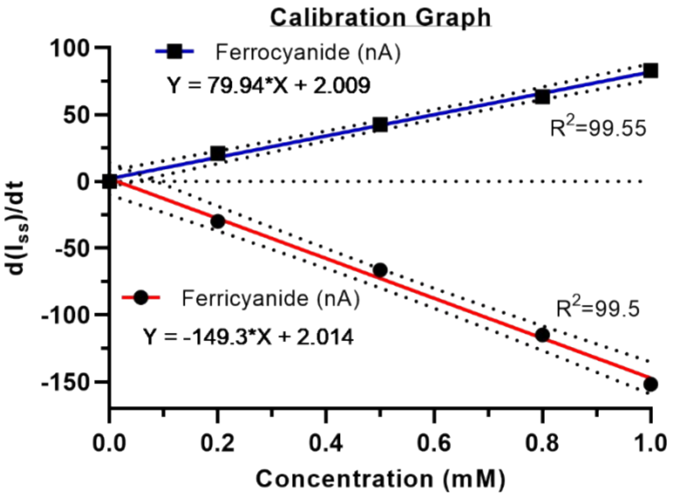

Figure S6. Calibration curve to determine Fe concentration. First derivative peaks from graphs in figures S5 and 3a against Ferricyanide or ferrocyanide concentration with line of best fit and 95% confidence limit.

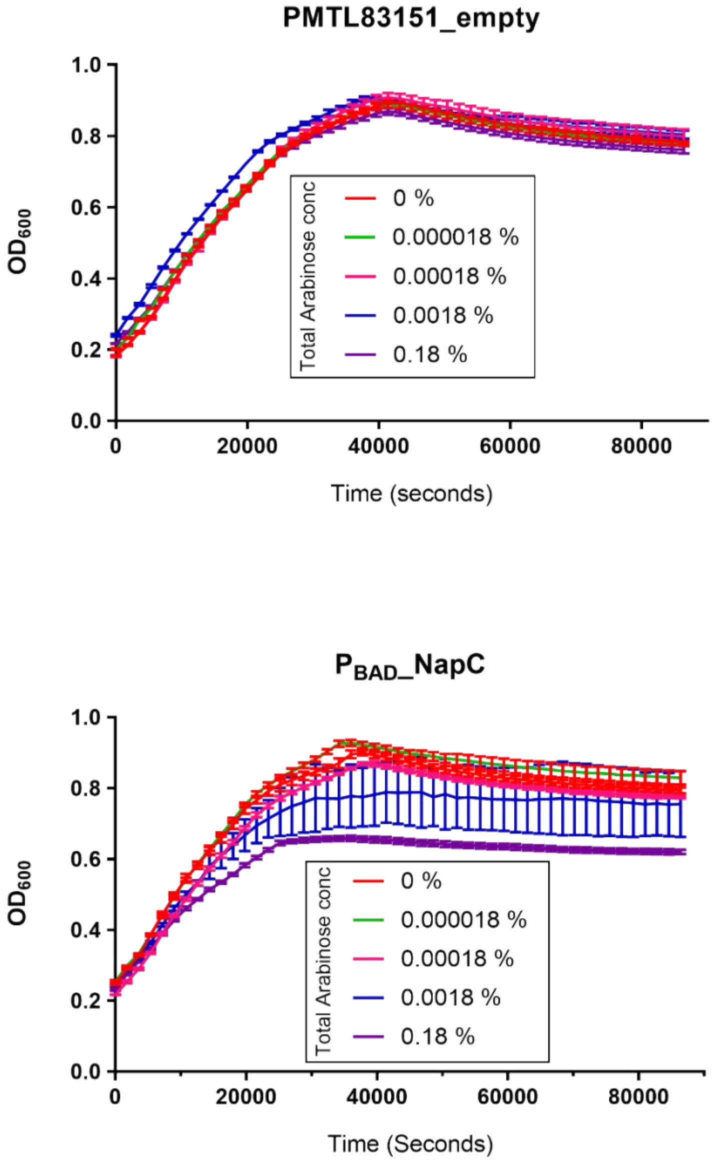

Figure S7. Arabinose toxicity study for, Top: *E. coli* containing empty plasmid and Bottom: *E. coli* containing Inducible vector Plasmid at different arabinose concentrations.
